# Supplementary material for: Sonic hedgehog signaling directs patterned cell remodeling during cranial neural tube closure
Source: eLife. 2020 Oct 26;9:e60234. doi: 10.7554/eLife.60234 (PMC7655103; doi:10.7554/eLife.60234)
Supplement: Supplementary file 1. — The details of the statistical tests performed, including the exact n and p values in the Figures and Figure supplements, are presented. [file elife-60234-supp1.docx]

**Supplementary File 1. N values and details of statistical analyses performed.**

| Figure | Panel | n | Mean ± SD | p | Statistical test and notes |
| --- | --- | --- | --- | --- | --- |
| 1 | G, H | 0 somites: 3 embryos  (565, 474, 488 cells)  4-6 somites: 6 embryos (857, 761, 825, 718, 667, 889 cells)  7-9 somites: 5 embryos (1000, 1270, 1198, 1047, 1377 cells) | 36.59 ± 2.20 μm^2^  24.86 ± 2.82 μm^2^  16.62 ± 2.16 μm^2^ | 0 vs. 4-6:  p = 0.0027  0 vs. 7-9:  p = 0.0006  4-6 vs. 7-9:  p = 0.0011 | Brown-Forsythe and Welch One-way ANOVA (Dunnett’s T3 multiple comparisons)  Does not assume equal SDs |
|  |  |  |  |  |  |
| 2 | B, C | 0-2 somites: 3 embryos (205, 158, 174 cells)  5 somites: 3 embryos  (205, 231, 197 cells)  7-9 somites: 3 embryos (192, 164, 166 cells) | 48.25 ± 4.99 μm^2^  41.22 ± 2.22 μm^2^  49.42 ± 3.32 μm^2^ | 0-2 vs. 5:  p = 0.2470  0-2 vs. 7-9:  p = 0.9781  5 vs. 7-9:  p = 0.0878 | Brown-Forsythe and Welch One-way ANOVA (Dunnett’s T3 multiple comparisons)  Does not assume equal SDs |
|  |  |  |  |  |  |
| 2 S1 | B | 0 somites: 3 embryos  (393, 341, 331 cells)  4-6 somites: 6 embryos (447, 595, 632, 517, 566, 484 cells)  7-9 somites: 5 embryos (552, 711, 979, 799, 915 cells) | ML: 49.81 ± 2.97%  AP: 50.19 ± 2.97%  ML: 69.15 ± 9.18%  AP: 30.85 ± 9.18%  ML: 78.17 ± 8.52%  AP: 21.83 ± 8.52% | 0 vs. 4-6:  p = 0.0085  0 vs. 7-9:  p = 0.0003  4-6 vs. 7-9:  p = 0.2228 | Two-way ANOVA (Sidak’s multiple comparisons) |
|  |  |  |  |  |  |
|  | C | 0 somites: 3 embryos  (393, 341, 331 cells)  4-6 somites: 6 embryos (447, 595, 632, 517, 566, 484 cells)  7-9 somites: 5 embryos (552, 711, 979, 799, 915 cells) | 1.10 ± 0.03  1.34 ± 0.15  1.49 ± 0.19 | 0 vs. 4-6:  p = 0.0233  0 vs. 7-9:  p = 0.0322  4-6 vs. 7-9:  p = 0.4490 | Brown-Forsythe and Welch One-way ANOVA (Dunnett’s T3 multiple comparisons)  Does not assume equal SDs |
|  |  |  |  |  |  |
|  | E | 0 somites: 3 embryos  (134, 109, 96 cells)  4-6 somites: 3 embryos  (132, 148, 131 cells)  7-9 somites: 3 embryos  (121, 107, 102 cells) | ML: 66.12 ± 10.41%  AP: 33.86 ± 10.41%  ML: 62.89 ± 16.14%  AP: 37.10 ± 16.14%  ML: 67.62 ± 15.89 %  AP: 32.37 ± 15.89% | 0 vs. 4-6:  p > 0.9999  0 vs. 7-9:  p > 0.9999  4-6 vs. 7-9:  p > 0.9999 | Two-way ANOVA (Sidak’s multiple comparisons) |
|  |  |  |  |  |  |
|  | F | 0 somites: 3 embryos  (134, 109, 96 cells)  4-6 somites: 6 embryos (132, 148, 131 cells)  7-9 somites: 5 embryos (121, 107, 102 cells) | 1.24 ± 0.16  1.28 ± 0.25  1.42 ± 0.31 | 0 vs. 5:  p = 0.9939  0 vs. 7-9:  p = 0.7590  5 vs. 7-9:  p = 0.8903 | Brown-Forsythe and Welch One-way ANOVA (Dunnett’s T3 multiple comparisons)  Does not assume equal SDs |
|  |  |  |  |  |  |
| 3 | B | 0-1 somite: 3 embryos  6-7 somites: 4 embryos  8-9 somites: 4 embryos | 1.34 ± 0.02  1.16 ± 0.02  0.95 ± 0.07 | 0-1 vs. 6-7:  p = 0.0023  0-1 vs. 8-9:  p < 0.0001  6-7 vs. 8-9:  p = 0.0004 | One-way ANOVA (Tukey’s multiple comparisons) |
|  |  |  |  |  |  |
|  | C | 0-1 somite: 3 embryos  6-7 somites: 4 embryos  8-9 somites: 4 embryos | 32.94 ± 3.59 μm  30.84 ± 2.18 μm  51.74 ± 3.21 μm | 0-1 vs. 6-7:  p = 0.6438  0-1 vs. 8-9:  p < 0.0001  6-7 vs. 8-9:  p < 0.0001 | One-way ANOVA (Tukey’s multiple comparisons) |
|  |  |  |  |  |  |
|  | D | 0-1 somite: 3 embryos  6-7 somites: 4 embryos  8-9 somites: 4 embryos | 18.38 ± 3.34 μm  18.68 ± 2.22 μm  30.40 ± 1.90 μm | 0-1 vs. 6-7:  p = 0.9858  0-1 vs. 8-9:  p = 0.0005  6-7 vs. 8-9:  p = 0.0004 | One-way ANOVA (Tukey’s multiple comparisons) |
|  |  |  |  |  |  |
|  | E | 0-1 somite: 3 embryos  6-7 somites: 4 embryos  8-9 somites: 4 embryos | 1.81 ± 0.13  1.67 ± 0.25  1.71 ± 0.20 | 0-1 vs. 6-7:  p = 0.6798  0-1 vs. 8-9:  p = 0.8234  6-7 vs. 8-9:  p = 0.9587 | One-way ANOVA (Tukey’s multiple comparisons) |
|  |  |  |  |  |  |
|  | G | 408 cells, 3 embryos (111, 169, 128 cells) | N/A | N/A | N/A |
|  |  |  |  |  |  |
|  | I | 60 cells, 3 embryos (20, 20, 20 cells) | N/A | N/A | N/A |
|  |  |  |  |  |  |
| 3 S1 | A | 0-1 somite: 3 embryos  6-7 somites: 4 embryos  8-9 somites: 4 embryos | 652.5 ± 95.9 μm  494.9 ± 33.4 μm  406.5 ± 32.5 μm | 0-1 vs. 6-7:  p = 0.0149  0-1 vs. 8-9:  p = 0.0011  6-7 vs. 8-9:  p = 0.1236 | One-way ANOVA (Tukey’s multiple comparisons) |
|  |  |  |  |  |  |
|  | B | 0-1 somite: 3 embryos  6-7 somites: 4 embryos  8-9 somites: 4 embryos | 486.5 ± 71.1 μm  425.6 ± 29.1 μm  427.5 ± 7.5 μm | 0-1 vs. 6-7:  p = 0.0976  0-1 vs. 8-9:  p = 0.1320  6-7 vs. 8-9:  p = 0.9998 | One-way ANOVA (Tukey’s multiple comparisons) |
|  |  |  |  |  |  |
| 3 S2 | B | 3 embryos (50, 50, 50 cells) | E1: 0.17 ± 1.13  E2: 0.60 ± 1.61  E3: 0.03 ± 0.99 | N/A | N/A |
|  |  |  |  |  |  |
| 4 | J | WT: 4 embryos (1415, 1367, 1186, 1240 cells)  *Ift122*: 4 embryos  (932, 906, 935, 757 cells) | 13.63 ± 0.82 μm^2^  21.06 ± 2.00 μm^2^ | p=0.0024 | Welch’s t-test  Does not assume equal SDs |
|  |  |  |  |  |  |
|  | K | WT: 5208 cells, 4 embryos    *Ift122*: 3530 cells, 4 embryos | N/A (D = 0.2763) | p<0.0001 | Kolmogorov-Smirnov |
|  |  |  |  |  |  |
|  | L | WT: 4 embryos (1171, 1162, 1116, 1148 cells)  *Ttc21b*: 3 embryos  (580, 687, 613 cells) | 15.60 ± 0.78 μm^2^  30.05 ± 2.39 μm^2^ | p=0.0058 | Welch’s t-test  Does not assume equal SDs |
|  |  |  |  |  |  |
|  | M | WT: 4597 cells, 4 embryos  *Ttc21b*: 1880 cells, 3 embryos | N/A (D = 0.4536) | p<0.0001 | Kolmogorov-Smirnov |
|  |  |  |  |  |  |
| 4 S4 | A | WT: 4 embryos (835, 1138, 1052, 990 cells)  *Ift122*: 4 embryos  (715, 867, 626, 563 cells) | WT ML: 72.32 ± 2.74%  WT AP: 27.68 ± 2.74%  *Ift122* ML: 65.14 ± 1.89%  *Ift122* AP: 34.86 ± 1.89% | WT vs. *Ift122* ML: p = 0.0020  WT vs. *Ift122* AP: p = 0.0020 | Two-way ANOVA (Sidak’s multiple comparisons) |
|  |  |  |  |  |  |
|  | B | WT: 4 embryos (835, 1138, 1052, 990 cells)  *Ift122*: 4 embryos (715, 867, 626, 563 cells) | 1.33 ± 0.03  1.22 ± 0.04 | p = 0.0058 | Welch’s t-test  Does not assume equal SDs |
|  |  |  |  |  |  |
|  | C | WT: 3 embryos (146, 170, 195 cells)  *Ift122*: 3 embryos (372, 599, 414 cells) | WT ML: 59.80 ± 15.49%  WT AP: 40.20 ± 15.49%  *Ift122* ML: 65.18 ± 11.60%  *Ift122* AP: 34.82 ± 11.60% | WT vs. *Ift122* ML: p = 0.8725  WT vs. *Ift122* AP: p = 0.8725 | Two-way ANOVA (Sidak’s multiple comparisons) |
|  |  |  |  |  |  |
|  | D | WT: 3 embryos  (146, 170, 195 cells)  *Ift122*: 3 embryos  (372, 599, 414 cells) | 1.20 ± 0.16  1.26 ± 0.12 | p = 0.6810 | Welch’s t-test  Does not assume equal SDs |
|  |  |  |  |  |  |
|  | E | WT: 4 embryos (913, 898, 870, 912 cells)  *Ttc21b*: 3 embryos  (415, 489, 411 cells) | WT ML: 72.36 ± 3.00%  WT AP: 27.64 ± 3.00%  *Ttc21b* ML: 59.99 ± 6.91%  *Ttc21b* AP: 40.01 ± 6.91% | WT vs. *Ttc21b* ML: p = 0.0167  WT vs. *Ttc21b* AP: p = 0.0167 | Two-way ANOVA (Sidak’s multiple comparisons) |
|  |  |  |  |  |  |
|  | F | WT: 4 embryos (913, 898, 870, 912 cells)  *Ttc21b*: 3 embryos  (415, 489, 411 cells) | 1.34 ± 0.09  1.22 ± 0.07 | p = 0.0863 | Welch’s t-test  Does not assume equal SDs |
|  |  |  |  |  |  |
| 4 S5 | E | WT:  3 embryos  *Ift122*:  3 embryos | WT 0-100:  2.34 ± 0.76 %  WT 100-200:  3.48 ± 0.50 %  WT 200-300: 4.12 ± 0.59 %  WT 300-400:  3.60 ± 0.72 %  *Ift122* 0-100:  2.37 ± 0.83 %  *Ift122* 100-200:  3.67 ± 0.59 %  *Ift122* 200-300: 4.05 ± 0.15 %  *Ift122* 300-400:  3.62 ± 0.75 % | WT vs. *Ift122*  0-100:  p > 0.9999  100-200:  p = 0.9950  200-300:  p > 0.9999  300-400:  p > 0.9999 | Two-way ANOVA (Sidak’s multiple comparisons) |
|  |  |  |  |  |  |
|  | F | WT:  3 embryos  *Ttc21b*:  3 embryos | WT 0-100:  3.25 ± 0.36 %  WT 100-200:  4.31 ± 0.48 %  WT 200-300: 4.22 ± 0.31 %  WT 300-400:  4.40 ± 0.38 %  *Ttc21b* 0-100:  2.78 ± 0.29 %  *Ttc21b* 100-200:  4.52 ± 0.16 %  *Ttc21b* 200-300: 4.25 ± 0.26 %  *Ttc21b* 300-400:  4.18 ± 0.90 % | WT vs. *Ttc21b*  0-100:  p = 0.6082  100-200:  p = 0.9633  200-300:  p > 0.9999  300-400:  p > 0.9618 | Two-way ANOVA (Sidak’s multiple comparisons) |
|  |  |  |  |  |  |
| 4 S6 | B | WT: 3 embryos  *Ttc21b*: 3 embryos | 77.00 ± 5.57 cells  75.33 ± 5.13 cells | p = 0.7225 | Welch’s t-test  Does not assume equal SDs |
|  |  |  |  |  |  |
| 5 | C, D | WT lateral: 4 embryos (data from Figure 4K)  WT midline: 3 embryos (276, 316, 285 cells)  *Ift122* lateral: 4 embryos (data from Figure 4K)  *Ift122* midline: 3 embryos (516, 441, 539 cells) | N/A | N/A | N/A |
|  |  |  |  |  |  |
|  | E | WT lateral: 4 embryos (data from Figure 4J)  WT midline: 3 embryos (276, 316, 285 cells)  *Ift122* lateral: 4 embryos (data from Figure 4J)  *Ift122* midline: 3 embryos (516, 441, 539 cells) | WT:  lateral: 13.63 ± 0.82 μm^2^  midline: 32.45 ± 2.18 μm^2^  *Ift122*:  lateral: 21.06 ± 2.00 μm^2^  midline: 19.23 ± 2.37 μ­m^2^ | WT lat vs. WT mid:  p = 0.0149  *Ift122* lat vs. *Ift122* mid:  p = 0.8383  WT lat vs. *Ift122* lat:  p = 0.0100  WT lat vs. *Ift122* mid:  p = 0.1723  WT mid vs. *Ift122* lat:  p = 0.0091  WT mid vs. *Ift122* mid:  p = 0.0090 | Brown-Forsythe and Welch One-way ANOVA (Dunnett’s T3 multiple comparisons)  Does not assume equal SDs |
|  |  |  |  |  |  |
|  | G | WT: 3 embryos  *Ift122*: 3 embryos | N/A | N/A | N/A |
|  |  |  |  |  |  |
|  | H | WT: 3 embryos  *Ttc21b*: 3 embryos | N/A | N/A | N/A |
|  |  |  |  |  |  |
|  | I | WT (lateral and midline): 3 embryos  *Ift122* (lateral and midline): 3 embryos | WT:  lateral: 59.80 ± 1.90 μm  midline: 26.63 ± 2.97 μm  *Ift122*:  lateral: 51.13 ± 1.10 μm  midline: 45.81 ± 3.96 μ­m | WT lat vs. WT mid:  p = 0.0019  *Ift122* lat vs. *Ift122* mid:  p = 0.4142  WT lat vs. *Ift122* lat:  p = 0.0239  WT lat vs. *Ift122* mid:  p = 0.0433  WT mid vs. *Ift122* lat:  p = 0.0034  WT mid vs. *Ift122* mid:  p = 0.0111 | Brown-Forsythe and Welch One-way ANOVA (Dunnett’s T3 multiple comparisons)  Does not assume equal SDs |
|  |  |  |  |  |  |
| 5 S1 | B | WT: 3 embryos  *Ttc21b*: 3 embryos | WT lateral: 44.77 ± 2.66 μm  WT midline: 21.40 ± 3.06 μm  *Ttc21b* lateral: 48.44 ± 3.32 μm  *Ttc21b* midline: 44.91 ± 6.12 μm | WT lat vs. WT mid: p = 0.0025  WT lat vs *Ttc21b* lat: p = 0.6285  WT lat vs. *Ttc21b* mid: p > 0.9999  WT mid vs. *Ttc21b* lat:  p = 0.0021  WT mid vs. *Ttc21b* mid:  p = 0.0352  *Ttc21b* lat vs. *Ttc21b* mid: p = 0.9140 | Brown-Forsythe and Welch One-way ANOVA (Dunnett’s T3 multiple comparisons)  Does not assume equal SDs |
|  |  |  |  |  |  |
|  | C | WT: 3 embryos  *Ttc21b*: 3 embryos | 420.0 ± 24.2 μm  601.3 ± 44.1 μm | p = 0.0075 | Welch’s t-test  Does not assume equal SDs |
|  |  |  |  |  |  |
|  | D | WT: 3 embryos  *Ttc21b*: 3 embryos | 1.00 ± 0.04  1.16 ± 0.04 | p = 0.0076 | Welch’s t-test  Does not assume equal SDs |
|  |  |  |  |  |  |
|  | E | WT: 3 embryos  *Ift122*: 3 embryos | 313.3 ± 14.2 μm  387.0 ± 47.9 μm | p = 0.1065 | Welch’s t-test  Does not assume equal SDs |
|  |  |  |  |  |  |
|  | F | WT: 3 embryos  *Ift122:* 3 embryos | 0.66 ± 0.07  0.92 ± 0.11 | p = 0. 0314 | Welch’s t-test  Does not assume equal SDs |
|  |  |  |  |  |  |
| 6 | B (*Ift122*) | WT:  86 cables, 3 embryos  *Ift122*:  36 cables, 3 embryos | WT circular mean: 24.2°  *Ift122*  circular mean: 41.5° | P < 0.05 | Watson nonparametric two-sample test for homogeneity |
|  |  |  |  |  |  |
|  | B (*Ttc21b*) | WT:  84 cables, 3 embryos  *Ttc21b:*  29 cables, 3 embryos | WT circular mean: 26.4°  *Ttc21b* circular mean: 44.3° | 0.05 < P < 0.10 | Watson nonparametric two-sample test for homogeneity |
|  |  |  |  |  |  |
|  | C (*Ift122)* | WT:  3 embryos  *Ift122*:  3 embryos | 28.67 ± 6.11 cables  12.33 ± 3.06 cables | p = 0.0266 | Welch’s t-test  Does not assume equal SDs |
|  |  |  |  |  |  |
|  | C (*Ttc21b*) | WT:  3 embryos  *Ttc21b*:  3 embryos | 28.0 ± 1.7 cables  9.7 ± 5.0 cables | p = 0.0160 | Welch’s t-test  Does not assume equal SDs |
|  |  |  |  |  |  |
|  | D (*Ift122*) | WT:  3 embryos  *Ift122*:  3 embryos | 50.7 ± 9.3 cables  33.3 ± 4.0 cables | p = 0.06668 | Welch’s t-test  Does not assume equal SDs |
|  |  |  |  |  |  |
|  | D (*Ttc21b*) | WT:  3 embryos  *Ttc21b*:  3 embryos | 43.4 ± 3.8 cables  33.7 ± 4.2 cables | p = 0.0414 | Welch’s t-test  Does not assume equal SDs |
|  |  |  |  |  |  |
|  | F (*Ift122*) | WT:  151 cables, 3 embryos  *Ift122*:  100 cables, 3 embryos | WT circular mean: 34.7°  *Ift122*  circular mean: 45.9° | P < 0.01 | Watson nonparametric two-sample test for homogeneity |
|  |  |  |  |  |  |
|  | F (*Ttc21b*) | WT:  130 cables, 3 embryos  *Ttc21b:*  101 cables, 3 embryos | WT circular mean: 28.8°  *Ttc21b* circular mean: 40.8° | P < 0.001 | Watson nonparametric two-sample test for homogeneity |
|  |  |  |  |  |  |
|  | G (*Ift122*) | WT: 3 embryos  (50, 50, 50 cells)  *Ift122*: 3 embryos  (50, 50, 50 cells) | N/A | N/A | N/A |
|  |  |  |  |  |  |
|  | H (*Ttc21b*) | WT: 3 embryos  (50, 50, 50 cells)  *Ttc21b*: 3 embryos  (50, 50, 50 cells) | N/A | N/A | N/A |
|  |  |  |  |  |  |
| 7 | B | WT: 3 embryos  *Ift122*: 3 embryos | N/A | N/A | N/A |
|  |  |  |  |  |  |
|  | C | WT: 3 embryos  *Ttc21b*: 3 embryos | N/A | N/A | N/A |
|  |  |  |  |  |  |
| 8 | B, C | WT: 5 embryos  (1129, 1011, 1047, 1269, 1105 cells)  *Gli2*: 5 embryos  (1075, 967, 1067, 1017, 1097 cells) | 17.39 ± 0.78 μm^2^  16.96 ± 1.57 μm^2^ | p = 0.6069 | Welch’s t-test  Does not assume equal SDs |
|  |  |  |  |  |  |
|  | E, F | WT: 5 embryos  (233, 220, 161, 342, 310 cells)  *Gli2*: 5 embryos  (228, 257, 277, 171, 174 cells) | 47.66 ± 7.20 μm^2^  32.24 ± 5.98 μm^2^ | p = 0.0066 | Welch’s t-test  Does not assume equal SDs |
|  |  |  |  |  |  |
|  | H | WT: 5 embryos  *Gli2*: 5 embryos | WT lateral:  59.54 ± 2.14 μm  WT midline:  29.90 ± 1.80 μm  *Gli2* lateral:  57.37 ± 5.95 μm  *Gli2* midline:  50.01 ± 3.04 μm | WT lat vs. WT mid: p < 0.0001  WT lat vs. *Gli2* lat: p = 0.9555  WT lat vs. *Gli2* mid: p = 0.0038  WT mid vs. *Gli2* lat: p = 0.0009  WT mid vs. *Gli2* mid: p < 0.0001  *Gli2* lat vs. *Gli2* mid: p = 0.2100 | Brown-Forsythe and Welch One-way ANOVA (Dunnett’s T3 multiple comparisons)  Does not assume equal SDs |
|  |  |  |  |  |  |
| 8S2 | A | WT: 5 embryos  (903, 796, 823, 792, 860 cells)  *Gli2*: 5 embryos  (1023, 867, 831, 738, 836 cells) | WT ML:  74.76 ± 5.95%  WT AP:  25.24 ± 5.95%  *Gli2* ML:  71.73 ± 7.47%  *Gli2* AP:  28.63 ± 7.47% | WT vs. *Gli2* ML: p = 0.6853  WT vs. *Gli2* AP: p = 0.6853 | Two-way ANOVA (Sidak’s multiple comparisons) |
|  |  |  |  |  |  |
|  | B | WT: 5 embryos  (903, 796, 823, 792, 860 cells)  *Gli2*: 5 embryos  (1023, 867, 831, 738, 836 cells) | 1.31 ± 0.10  1.27 ± 0.13 | p = 0.6079 | Welch’s t-test  Does not assume equal SDs |
|  |  |  |  |  |  |
|  | C | WT: 5 embryos  (260, 173, 88, 105, 100 cells)  *Gli2*: 5 embryos  (244, 325, 180, 177, 146 cells) | WT ML:  66.59 ± 12.01%  WT AP:  33.41 ± 12.01%  *Gli2* ML:  66.99 ± 20.28%  *Gli2* AP:  33.01 ± 20.28% | WT vs. *Gli2* ML: p = 0.9991  WT vs. *Gli2* AP: p = 0.9991 | Two-way ANOVA (Sidak’s multiple comparisons) |
|  |  |  |  |  |  |
|  | D | WT: 5 embryos (260, 173, 88, 105, 100 cells)  *Gli2*: 5 embryos (244, 325, 180, 177, 146 cells) | 1.31 ± 0.20  1.38 ± 0.37 | p = 0.7324 | Welch’s t-test  Does not assume equal SDs |
|  |  |  |  |  |  |
| 9 | H | control: 3 embryos  (1140, 1104, 1395 cells)  Wnt1-Cre2 > SmoM2:  3 embryos (763, 867, 723 cells) | 15.15 ± 1.61 μm^2^  22.69 ± 2.07 μm^2^ | p = 0.0089 | Welch’s t-test  Does not assume equal SDs |
|  |  |  |  |  |  |
|  | I | control:  3639 cells, 3 embryos  Wnt1-Cre2 > SmoM2:  2353 cells, 3 embryos | N/A (D = 0.3002) | p < 0.0001 | Kolmogorov-Smirnov |
|  |  |  |  |  |  |
|  | J | control: 3 embryos  (225, 183, 189 cells)  Wnt1-Cre2 > SmoM2:  3 embryos (188, 176, 224 cells) | 43.76 ± 4.85 μm^2^  43.56 ± 3.81 μm^2^ | p = 0.9556 | Welch’s t-test  Does not assume equal SDs |
|  |  |  |  |  |  |
|  | K | control:  597 cells, 3 embryos  Wnt1-Cre2 > SmoM2:  588 cells, 3 embryos | N/A (D = 0.0320) | p = 0.9215 | Kolmogorov-Smirnov |
|  |  |  |  |  |  |
| 9 S1 | B | control:  4 embryos  Wnt1-Cre2 > SmoM2:  4 embryos | 3.73 ± 0.38 %  3.64 ± 0.44 % | p = 0.7798 | Welch’s t-test  Does not assume equal SDs |
|  |  |  |  |  |  |
| 9S2 | A | control: 3 embryos  (887, 912, 1132 cells)  SmoM2: 3 embryos  (567, 655, 753 cells) | control ML:  69.99 ± 3.13%  control AP:  30.01 ± 3.13%  SmoM2 ML: 69.19 ± 3.72%  SmoM2 AP:  30.81 ± 3.72% | control vs. SmoM2 ML:  p = 0.9519  control vs. SmoM2 AP:  p = 0.9519 | Two-way ANOVA (Sidak’s multiple comparisons) |
|  |  |  |  |  |  |
|  | B | control: 3 embryos  (887, 912, 1132 cells)  SmoM2: 3 embryos  (567, 655, 753 cells) | 1.29 ± 0.05  1.29 ± 0.60 | p = 0.9359 | Welch’s t-test  Does not assume equal SDs |
|  |  |  |  |  |  |
|  | C | control: 3 embryos  (85, 143, 125 cells)  SmoM2: 3 embryos  (146, 78, 144 cells) | control ML: 66.77 ± 14.70%  control AP: 33.23 ± 14.70%  SmoM2 ML: 66.01 ± 19.55%  SmoM2 AP: 33.99 ± 19.55% | control vs SmoM2  ML: p = 0.9983  control vs SmoM2 AP:  p = 0.9983 | Two-way ANOVA (Sidak’s multiple comparisons) |
|  |  |  |  |  |  |
|  | D | control: 3 embryos  (85, 143, 125 cells)  SmoM2: 3 embryos  (146, 78, 144 cells) | 1.31 ± 0.20  1.38 ± 0.37 | p = 0.7324 | Welch’s t-test  Does not assume equal SDs |
